# Differential impact of stay‐at‐home orders on mental health in adults who are homeschooling or “childless at home” in time of COVID‐19

**DOI:** 10.1111/famp.12698

**Published:** 2021-08-02

**Authors:** Esther Cuadrado, Alicia Arenas, Manuel Moyano, Carmen Tabernero

**Affiliations:** ^1^ Maimonides Biomedical Research Institute of Cordoba (IMIBIC) Cordoba Spain; ^2^ University of Cordoba Cordoba Spain; ^3^ University of Seville Seville Spain; ^4^ University of Salamanca Salamanca Spain; ^5^ Neurosciences Institute of Castilla y Leon (INCyL) Salamanca Spain

**Keywords:** Loneliness, Anxiety, Stress, Perceived social support provided by school staff, Homeschooling stress, COVID‐19

## Abstract

The COVID‐19 pandemic has forced the confinement of most populations worldwide, through stay‐at‐home orders. Children have continued their education process at home, supervised by parents, who, in most cases, have adopted the role of prime drivers of their learning processes. In this study, the psychological impact of confinement was explored, as well as the relationship of the forced homeschooling situation with psychological well‐being. During their confinement, 400 individuals residing in Spain—165 without children at home (Group 1), 104 parents who dedicated little time to homeschooling (Group 2), and 131 who dedicated more time to homeschooling (Group 3)—responded to an online questionnaire. The results show that confinement threatened the mental health of all the participants but especially Group 3 individuals, who had the highest loneliness, anxiety, and stress levels. Moreover, loneliness, perception of discomfort due to homeschooling, and anxiety exacerbated the stress experienced during confinement. Discomfort due to the homeschooling situation was especially relevant in explaining anxiety and stress for Group 3 individuals. These results suggest that forced homeschooling could be associated with the negative consequences that confinement has on individuals’ mental health. Moreover, the results suggest that parents who dedicate more time to homeschooling feel more unprotected and more stressed due to the homeschooling in comparison to Group 2 individuals. Health professionals must pay special attention to parents who dedicate more time to homeschooling, and governments and schools must emphasize social support provision to families during homeschooling situations.

## Resumen

La pandemia de la COVID‐19 ha impuesto el confinamiento de la mayoría de las poblaciones de todo el mundo mediante órdenes de quedarse en casa. Los niños han continuado su proceso de escolarización en sus hogares, supervisados por los padres, quienes, en la mayoría de los casos, han adoptado el papel de impulsores principales de sus procesos de aprendizaje. En este estudio se analizó el efecto psicológico del confinamiento, así como la relación de la situación forzada de enseñanza en el hogar con el bienestar psicológico. Durante su confinamiento, 400 personas residentes en España —165 sin niños en casa (grupo 1), 104 padres que dedicaron poco tiempo a la enseñanza en el hogar (grupo 2), y 131 que dedicaron más tiempo a la enseñanza en el hogar (grupo 3)— respondieron un cuestionario en línea. Los resultados demuestran que el confinamiento amenazó la salud mental de todos los participantes, pero especialmente la de las personas del grupo 3, quienes demostraron mayores niveles de soledad, ansiedad y estrés. Además, la soledad, la percepción de incomodidad a consecuencia de la enseñanza en el hogar, y la ansiedad agravaron el estrés sufrido durante el confinamiento. La incomodidad provocada por la situación de enseñanza en el hogar fue especialmente importante para explicar la ansiedad y el estrés de las personas del grupo 3. Estos resultados sugieren que la enseñanza forzada en el hogar podría estar asociada con las consecuencias negativas que tiene el confinamiento en la salud mental. Además, los resultados indican que los padres que dedican más tiempo a la enseñanza en el hogar se sienten más desprotegidos y más estresados debido a este tipo de enseñanza en comparación con las personas del grupo 2. Los profesionales de la salud deben prestar especial atención a los padres que dedican más tiempo a la enseñanza en el hogar, y los gobiernos y centros educativos deben brindar más apoyo social a las familias durante las situaciones de enseñanza en el hogar.

## 摘要

2019新冠病毒(COVID‐19)大流行病迫使世界各地的大多数人因为留在家里的命令被禁闭。孩子们在家里继续他们的教育过程，由父母监督，在大多数情况下，父母是他们学习过程的主要推动者。在这项研究中，我们探讨了禁闭的心理影响，以及强迫式的家庭教育境况与心理健康的关系。在禁闭期间，居住在西班牙的400名受试者‐‐其中165名家中没有孩子的人（第1组），104名家长很少有时间致力于家庭教育的父母（第2组），以及131名花较多时间致力于家庭教育的父母（第3组）‐‐回答了一份在线调查问卷。结果显示，禁闭威胁着所有参与者的心理健康，但特别是第3组的人，他们的孤独感、焦虑和压力水平最高。此外，孤独感、对家庭教育带来的不适感和焦虑感加剧了禁闭期间的压力。在解释第3组人的焦虑和压力时，由于家庭教育情况造成的不适感尤为重要。这些结果表明，强迫家庭教育可能与禁闭对个人心理健康产生的消极后果有关。此外，研究结果表明，与第2组个体相比，为家庭教育投入更多时间的父母感到更多的不受保护和更多的压力。卫生专业人员必须特别关注那些花更多时间在家庭教育上的父母，而政府和学校必须强调在在家上学的家庭教育情况下向家庭提供社会支持。

## INTRODUCTION

As of the date of this writing (June 24, 2020), a total of 9,266,021 cases of the new coronavirus SARS‐CoV‐2 have been confirmed in the world—246,752 in Spain (Coronavirus Resource Center, 2020), the country in which this study was performed. SARS‐CoV‐2 has obligated most countries worldwide to adopt containment and mitigation measures (Cohen & Kupferschmidt, 2020). Populations have been confined at home, with the closure or cancellation of national events, educational centers, facilities, and shops, as well as restrictions on national and international movement (Cohen & Kupferschmidt, 2020). In Spain—one of the hardest hit European countries—on March 14, the government declared a state of alarm, with the forced confinement of the population through a “stay‐at‐home” order and the closure of educational centers. These kinds of measures, resulting in extreme social distancing, have been effective in reducing the number of infections and deaths but have also had an impact on mental health, leading to feelings of loneliness, anxiety, stress, and depression, among other (Brooks et al., 2020; Hagger et al., 2020; Tull et al., 2020).

For many families, confinement implies that children must continue with their educational process through homeschooling. The obligation for parents to adopt the unexpected role of teacher in a forced homeschooling situation, in an attempt to slow down the effect of the unexpected educational disruption, is one more stressor that may have repercussions on mental health. Moreover, most individuals have had to continue working, with the added difficulty of reconciling work with the new forced role of the teacher. Although the psychological impact of the pandemic has been largely studied on the general population, there are still relatively few studies focused on parents and their adaptation to homeschooling. This study aims to explore the psychological impact of confinement for the Spanish population, and particularly in the population with children at home who are or are not involved with homeschooling.

### Loneliness, anxiety, and stress in confined individuals

Because different studies have found a psychological impact from stay‐at‐home measures (Brooks et al., 2020; Hagger et al., 2020; Röhr et al., 2020; Tull et al., 2020), it is expected that loneliness, anxiety, and stress could increase during confinement. Lockdown orders could have an impact on feelings of loneliness, with isolation preventing a real connection with others, especially with the closest people with whom individuals share a sense of belonging (e.g., friends, families, and colleagues). Humans are social in nature, and belongingness is a relevant need for individuals (Baumeister & Leary, 1995). Thus, forced isolation—which prevents the expression of group membership—could boost the incidence of loneliness as well as anxiety and stress. In this sense, it has been demonstrated that being quarantined produces stress, posttraumatic stress, and psychological distress (Bai et al., 2004; Liu et al., 2012), anxiety (Bai et al., 2004; Tull et al., 2020), and loneliness (Röhr et al., 2020; Tull et al., 2020).

Nevertheless, as far as we know, no investigation has studied the potentially different impact of social isolation measures in individuals without children at home and individuals with children at home. Some studies have found that the mental distress of parents increases during the pandemic (Gassman‐Pines et al., 2020; Janssen et al., 2020; Patrick et al., 2020), but those studies have not compared this increment with the increase of distress potentially suffered by people without children. Moreover, those studies have not explored the impact of forced homeschooling due to the lockdown situation. Framed in Folkman's theoretical propositions (Folkman, 2013), homeschooling may present an additional stressor for families, who must face an unexpected situation that requires them to adopt a role for which they have not necessarily been prepared, which can make them perceive that they do not have the necessary resources to cope with it effectively. Consequently, the enforcement of homeschooling could generate high levels of stress. Additionally, the adoption of this new role could create high levels of anxiety. Thus, stress and anxiety could be lower in individuals without children at home than in individuals who are involved with homeschooling. Moreover, parents who spend more time with homeschooling could perceive higher anxiety and stress than those who spend little or no time with homeschooling.

But what about the loneliness of people with and without children at home during confinement? In an unprecedented situation of social isolation, having children at home should protect individuals from loneliness. In this sense, previous studies have demonstrated that adults without children feel more lonely than adults with children (Beutel et al., 2017).

### Homeschooling as a stressful situation

Another variable to consider regarding parents with school‐age children at home during stay‐at‐home orders is the appraisal of discomfort due to the experience of homeschooling. The lockdown derived from the pandemic has changed the educational experience, being remote and online rather than face to face (Verma et al., 2020). This new way of teaching may also affect the perception of social support provided by teachers and school staff (SSbySchools) and the stress directly associated with parents’ new role (homeschooling stress). In fact, parents’ capacity to effectively provide and use the different structures and resources available for the new homeschooling situation and to cope effectively with homeschooling may vary greatly from one parent to another (Doyle, 2020; Oreopoulos et al., 2003). Furthermore, in this forced homeschooling situation, SSbySchools becomes increasingly necessary. In this sense, parents who perceive a lack of SSbySchools may increase their dedication to homeschooling, in an attempt to supply the missing support from schools; thus, we might be expected to find higher levels of perceived lack of SSbySchools in parents who dedicate more time to homeschooling in comparison to those who spend little or no time on homeschooling. Moreover, considering that even parents who have chosen homeschooling in nonpandemic circumstances often suffer from “homeschooling burnout” (Lois, 2006, 2010), it is easy to think that the forced situation of homeschooling may induce parents to homeschooling stress. In this sense, homeschooling may result in a stressful event, especially for those who spend significant daily time taking care of homeschooling.

### Explaining stress and anxiety during confinement

As argued above, situations that imply social isolation, such as confinement, may affect feelings of loneliness, stress, and anxiety (Bai et al., 2004; Liu et al., 2012; Tull et al., 2020). Also, the literature has established relationships among these factors. Loneliness affects mental health (Beutel et al., 2017; Heinrich & Gullone, 2006) by producing anxiety (Beutel et al., 2017; Kara et al., 2019; Muyan et al., 2016; Zawadzki et al., 2013), among other emotional outcomes. Thus, it is expected that more the people feel lonely during confinement, the more they would experience anxiety.

Moreover, there is a clear relationship between anxiety and stress, and this can be directed in both ways: stress as a predictor or a determinant of anxiety. Different studies have found that stressful situations may produce anxiety disorders (Campos et al., 2013; McEwen et al., 2012; Zvolensky et al., 2002). Others have explained how the anxiety level displayed by individuals (which may be due to stressful situation exposure) interferes with the capacity to cope effectively with stress, being then related to acute stress levels (Fawzy & Hamed, 2017; Gillott & Standen, 2007; Phillips et al., 2015). Stress generation and stress causation reflect the fact that the links between anxiety and stress may be bidirectional. Thus, in a stressful confinement situation it can be expected that high anxiety could reduce the capacity to cope with stress, and thus cause a higher stress level, and also that high stress produced by confinement could make individuals more sensitive to anxiety.

Additionally, the appraisal of discomfort due to the experience of homeschooling is relevant in the experience of anxiety and stress during confinement. Effective social support has been consistently related to good mental health, including low rates of anxiety (Fasihi Harandi et al., 2017) and stress (Çivitci, 2015). Thus, parents who perceive a lack of SSbySchools during the homeschooling experience could perceive more anxiety and stress during confinement. Also, the homeschooling stress could influence anxiety and general stress during confinement. Lois (2006, 2010) has shown that voluntarily chosen homeschooling could produce stress in individuals. Thus, forced homeschooling due to confinement could interfere with the resource capacity perceived by individuals to manage the situation, resulting in more acute stress and anxiety.

### Purpose of the study

The main aim of this study was to explore the psychological ramifications of confinement in individuals by comparing three population groups: those without children at home, those with children at home who are involved with homeschooling, and those who are not involved with homeschooling. Considering the reviewed literature, the following study hypotheses (H) are proposed:

- H1. Anxiety and stress are (a) higher during confinement than before confinement and (b) higher during confinement in people who spend more time with homeschooling than in people who spend little or no time with homeschooling, and people without children at home.
- H2. Loneliness levels are (a) higher during confinement than before confinement and (b) higher both before and during confinement in people without children at home compared to people with children at home.
- H3. The perception of lack of SSbySchools and homeschooling stress is higher in people who dedicate more time to homeschooling than in people who spend little or no time on homeschooling.
- H4. Different relations among loneliness, anxiety, and stress exist. Specifically, it is hypothesized that (a) loneliness suffered during confinement is related to anxiety and (b) anxiety and stress levels during confinement are related to each other.
- H5. The perception of poor SSbySchools and high homeschooling stress is related to the stress and anxiety levels during confinement.

## METHOD

### Procedure

On April 25, 2020, by using a combination of convenience and snowball sampling methods, the researchers shared a link to an online questionnaire among their social networks and with different associations of parents of students throughout Spain. Both participation and diffusion of the questionnaire were solicited. Informed consent was obtained. The study was conducted in accordance with the Declaration of Helsinki and was not reviewed by any ethics committee (the Spanish Ministry of Science and Innovation requires such approval only when studies involve human or animal experimentation). Respondents completed the questionnaire during confinement, in late April 2020 (82.5% of respondents) and the first two weeks of May 2020 (17.5% of respondents). The Spanish population was confined as of March 14, and the confinement did not end before mid‐June. Thus, the participants responded to the questionnaire about 4–6 weeks after the starting point of the confinement situation and in a confinement period in which the COVID‐19 cases and deaths were declining.

### Participants

The sample consisted of 400 participants residing in Spain during the study period. Depending on whether they had children living with them and whether they were involved with homeschooling during this time, the sample was divided into three groups: Group 1 (G1) comprised 165 individuals without children at home during this time; G2 comprised 104 individuals with children at home and who spent little or no time on homeschooling (1 h or less per day); and G3 comprised 131 individuals with children at home and who spent more time with homeschooling (2 h or more). The cut points of “less than 1 h per day” and “2 h per day or more” were selected because the aim was to observe and compare parents who spent little or no time versus parents who spent more time with homeschooling. Meanwhile, 1 h per day can seem rather low in comparison to the 5 h per typical school day for Spanish children. The mean of homeschooling in our data was only 131.27 min per day (SD = 129.57), and 44.3% of the parents with children at home (almost half of the parent sample) spent 1 h or less per day with homeschooling. Moreover, the rather low cut‐point of 1 h or less per day allowed us to determine a group with a low level of involvement in homeschooling versus a group with higher dedication. Regarding the distribution of reported homeschooling dedication, for G2, 53.8% reported no dedication to homeschooling, 25% reported only 30 min per day, and 21.2% reported 1 h of dedication per day; and for G3, 25.2% reported 2 h of dedication, 27.5% reported 3 h, 19.1% reported 4 h, 14.5% reported 5 h, 7.6% reported 6 h, and 13.2% reported a dedication of 7 or more hours per day. In participants with children at home, women (141.59 min, SD = 132.54) dedicated marginally (*p* = .056) more time to homeschooling than did men (105.92 min, SD = 119.12). The sociodemographic characteristics can be observed in Table 1.

**TABLE 1:** Socio‐demographic characteristics of the participants

	Global sample			G_1_			G_2_			G_3_			X2 (for %)/*t* (for M)		
	*N*	Valid %	M (SD)	*N*	Valid %	M (SD)	*N*	Valid %	M (SD)	*N*	Valid %	M (SD)	G1–G2	G1–G3	G2–G3
Gender															
Women	284	71.0	–	117	70.9	–	68	65.4	–	99	75.6	–	1.69	0.81	2.93
Men	116	29.0	–	48	29.1	–	36	34.6	–	32	24.4	–			
Age	–	–	43.02 (11.51)	–	–	40.93 (16.00)	–	–	47.39 (6.97)	–	–	42.19 (4.90)	−3.89***	−0.87	6.71***
Place of residence															
Andalusia	275	68.8	–	137	83.0	–	71	68.3	–	67	51.1	–	7.93**	34.66***	7.01**
Castile and Leon	76	19.0	–	4	2.4	–	20	19.2	–	52	39.7	–	22.17***	66.13***	11.42**
Madrid	9	2.3	–	3	1.8	–	3	2.9	–	3	2.3	–	0.33	0.08	0.08
Catalonia	8	2.1	–	6	3.6	–	1	1.0	–	1	0.8	–	1.81	2.61	0.03
Aragon	8	2.0	–	4	2.4	–	1	1.0	–	3	2.3	–	0.75	0.01	0.61
Murcia	5	1.3	–	2	1.2	–	2	1.9	–	1	0.8	–	0.22	0.15	0.62
Estremadura	4	1.0	–	2	1.2	–	1	1.0	–	1	0.8	–	0.04	0.15	0.03
Elsewhere in Spain	15	4.3	–	27	4.4	–	5	4.7	–	3	2.2	–	0.05	0.85	1.12
Job situation															
Worker or student	270	69.0	–	104	63.7	–	73	70.9	–	93	74.4	–	1.45	2.08	0.02
Employed worker	221	56.5	–	82	50.3	–	62	60.2	–	77	61.6	–	–	–	–
Autonomous worker	34	8.7	–	11	6.7	–	11	10.7	–	12	9.6	–	–	–	–
Student	15	3.8	–	11	6.7	–	0	0.0	–	4	3.2	–	–	–	–
Jobless	121	31.0	–	59	36.2	–	30	29.1	–	32	25.6	–	1.38	4.40*	0.58
Unemployed	93	23.8	–	35	21.5	–	27	26.2	–	31	24.8	–	–	–	–
Retired	28	7.2	–	24	14.7	–	3	2.9	–	1	0.8	–	–	–	–
Participants reporting having a partner															
Yes	338	84.5	–	123	74.5	–	99	95.2	–	116	88.5	–	18.86***	9.21**	3.29
No	62	15.5	–	42	25.5	–	5	4.85	–	15	11.5	–			
Participants reporting caring for dependent person at home															
Person with disability	15	3.8	–	3	1.8	–	5	4.8	–	7	5.3	–	1.98	2.78	0.03
Dependent elderly	20	5	–	9	5.5	–	6	5.8	–	5	3.8	–	0.01	0.44	0.50
Average of individuals living at home and children living at home															
Number of individuals	–	–	3.15 (1.16)	–	–	2.39 (1.08)	–	–	3.57 (0.80)	–	–	3.78 (0.94)	−9.61***	−11.65***	−1.77
Number of children	–	–	1.02 (1.01)	–	–	0.00 (0.00)	–	–	1.59 (0.71)	–	–	1.84 (0.68)	−28.93***	−34.91***	−2.79**
0	165	41.3	–	165	100	–	0	0	–	0	0	–	–	–	–
1	95	23.8	–	–	–	–	54	51.9	–	41	31.3	–	–	–	–
2	112	28.0	–	–	–	–	41	39.4	–	71	54.2	–	–	–	–
3	25	6.3	–	–	–	–	7	6.7	–	18	13.7	–	–	–	–
4	3	.08	–	–	–	–	2	1.9	–	1	0.8	–	–	–	–
Participants reporting being single parent (families with only one parent at charge) and large families (families with three or more children)															
Single parent	59	14.8	–	–	–	–	17	16.3	–	20	15.3	–	0.72	0.29	0.11
Large families	47	11.8	–	–	–	–	12	11.5	–	25	19.1	–	2.83	12.13***	2.51
Minutes dedicated to HS	–	–	77.12 (118.46)	–	–	0.00 (0.00)	–	–	12.78 (24.58)	–	–	225.34 (97.91)	−6.68***	−29.58***	−21.60***

Note

G_1_ = Individuals without children at home; G_2_ = Individuals who spent little or no time with homeschooling; G_3_ = Individuals who spent more time with homeschooling; HS = Homeschooling.

### Measures

#### Appraisal of discomfort due to the homeschooling experience

To measure the extent to which individuals perceived poor SSbySchools and felt stress related to homeschooling, the Appraisal of Discomfort due to the Experience of Homeschooling Scale (ADEHS) was created. The scale, which can be provided by the authors upon request, consisted of six items (three for perceived lack of SSbySchools and three for homeschooling‐related stress). Exploratory factor analysis (EFA) performed with varimax rotation (Sample 1: 105 randomly selected participants with children at home) identified the expected two factors (Factor 1: lack of social support due to homeschooling [α = 0.89]; Factor 2: homeschooling stress [α = 0.91]) that explained jointly 84.82% of the variance, with all the items loading in the expected factor. Confirmatory factor analysis (Sample 2: the remaining 130 participants with children at home) provided evidence for the bifactorial structure, indicating the model good fit indices: χ^2^(6) = 6.573, *p* = 0.362; goodness of fit index (GFI) = 0.98; comparative fit index (CFI) = 0.99; and root mean square error of approximation (RMSEA) with 95% confidence interval = 0.027 (0.001, 0.120).

#### Loneliness

A brief version (three items) of the Revised University of California Los Angeles Loneliness Scale (R‐UCLA Loneliness Scale; Russell et al., 1980) was completed twice by participants. In the first iteration they assessed how much they felt lonely before the confinement, and in the second iteration how much they felt lonely during the confinement. Reliability was high for both iterations (α_before_ = 0.81; α_during_ = 0.79). The items chosen for the short version were the three items of the original scale with higher loading in an EFA carried out by our team in a previous unpublished pilot study.

#### Stress

To measure the extent to which individuals suffered from stress, the stress scale of the Spanish Labor, Environment and Health Trade Union Institute (ISTAS) promoted by the Superintendency of Social Security (SUSESO), in its 21st version (SUSESO‐ISTAS 21; Moncada et al., 2014), was completed twice by participants. Reliability was high for both iterations (α_before_ = 0.92; α_during_ = 0.93).

#### Anxiety

Anxiety was assessed using the validated Spanish version of the Hospital Anxiety and Depression Scale (HADS; Herrero et al., 2003), which was completed twice by participants. Reliability was high for both iterations (α_before_ = 0.76; α_during_ = 0.85).

### Design and data analyses

Changes in the variables were assessed with repeated measures analyses; pairwise comparisons were examined with the Bonferroni correction method to observe differences between groups and differences between before and during the confinement, depending on the group. To compare G2 and G3 in terms of level of perceived SSbySchools and homeschooling stress, analysis of variance (ANOVA) was performed.

The relationships of the studied variables with both anxiety and stress, as well as the percentage of variance explained by them, were explored by performing linear regression analyses (LRA) with two models. In the first model, the independent variables (IVs) inserted were those that were valid for the three samples (loneliness and stress or anxiety, depending on whether the dependent variable [DV] was anxiety or stress). In the second model, the variables that were valid uniquely for the two samples with homeschooling were added (lack of SSbySchools and homeschooling stress). Thus, only one model was examined for G1 but two models for G2 and G3. Changes in *R* ^2^ and *F* were inspected. To control for the effect of the IV, some sociodemographic variables (age and gender) were included in the equation as covariates.

To confirm the different implications of the homeschooling variables on stress and anxiety, depending on the time dedicated to homeschooling, multigroup analyses were performed using AMOS software. Because of the cross‐sectional nature of our study, two different competing models were explored by inversing the sense of the relation between stress and anxiety. In the first one the DV was anxiety, whereas in the second one it was stress. The goodness of the fits of each model was evaluated by checking the following parameters: chi‐square (χ^2^), χ^2^/*df*, root mean squared error of approximation (RMSEA), comparative fit index (CFI), goodness of fit index (GFI), adjusted goodness of fit index (AGFI), normed fit index (NFI), Tucker–Lewis index (TLI), and Akaike information criterion (AIC). Moreover, for each model (stress and anxiety), two different nested models were compared: in the first one (Model 0) all the hypothesized links were included, whereas in the second one (Model 1) the paths that were nonsignificant for both G2 and G3 were removed. If the difference in χ^2^ was significant between the nested models, the more parsimonious model (Model 0 or Model 1) of stress and of anxiety was selected by observing the parsimony‐adjusted measures (RMSEA and AIC). Once the more parsimonious model of stress and of anxiety had been selected, the choice of one over the other was made by comparing the parsimony fit indices (RMSEA and AIC) for each one of the models.

## RESULTS

### Differences by time and group in study variables: assessment of Hypotheses 1 and 2

**FIGURE 1:**
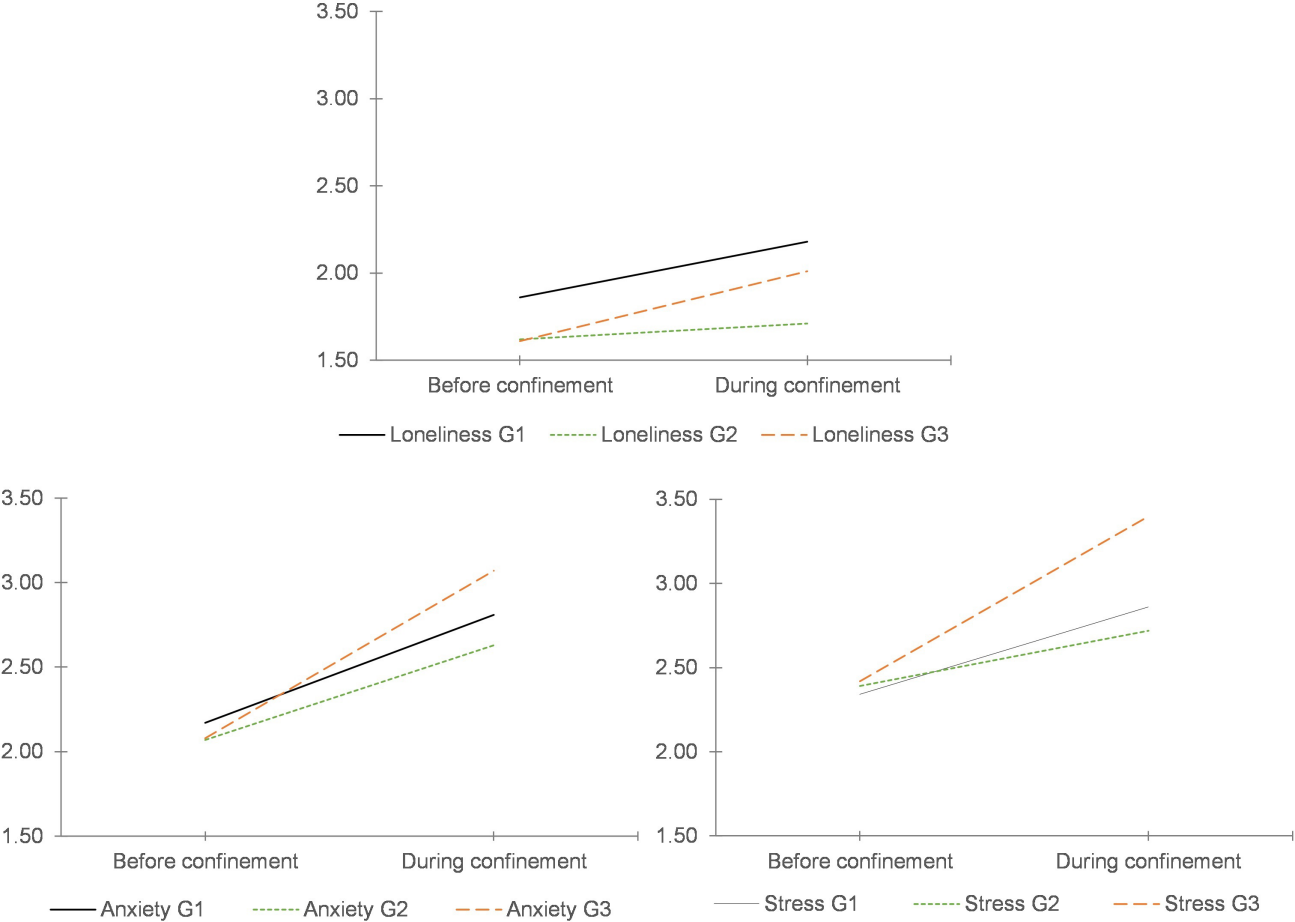
Change in loneliness, negative affect, stress, and anxiety before and during confinement. G1 = Individuals without children; G2 = Individuals who spent little or no time with homeschooling; G3 = Individuals who spent more time with homeschooling

As Figure 1 shows, significant changes were found between before and during confinement for each of the studied variables: loneliness: *F*(1, 397) = 48.183, *p* < 0.001, η^2^ = 0.108, observed power (OP) = 1.00; stress: *F*(1, 397) = 91.433, *p* < 0.001, η^2^ = 0.187, OP = 1.00; anxiety: *F*(1, 397) = 251.020, *p* < 0.001, η^2^ = 0.387, OP = 1.00. Thus, H1a and H2a were supported for the general sample: confinement increased perceptions of stress, anxiety (H1a), and loneliness (H2a) relative to pre‐confinement. Additionally, an interactive effect between living without children at home/spending or not spending time on homeschooling and each of the studied variables was found: loneliness: *F*(2, 397) = 5.091, *p* = 0.007, η^2^ = 0.025, OP = 0.820; stress: *F*(2, 397) = 8.640, *p* < 0.001, η^2^ = 0.042, OP = 0.968; anxiety: *F*(2, 397) = 7.594, *p* = 0.001, η^2^ = 0.037, OP = 0.945.

As seen in Table 2, the pairwise comparisons supported H1a (confinement increased perceptions of stress and anxiety) and H1b (perceptions of stress and anxiety during the confinement were higher in G3 than in G2). Nevertheless, H2a and H2b were only partially supported: the change in perceptions of loneliness (H2a) was statistically significant for G1 and G3, as expected, but not for G2; and differences between groups (H2b) during confinement were found between G2 and both G1 and G3, but the expected differences between G3 and G1 were not found.

**TABLE 2:** Mean differences in the studied variables between the different groups and comparing before and during confinement

	Loneliness		Stress		Anxiety	
	∆M	*p*	∆M	*p*	∆M	*p*
Mean difference, before and during confinement						
Group 1	**−0.327**	**<0.001**	**−0.516**	**<0.001**	**−0.644**	**<0.001**
Group 2	−0.090	0.238	**−0.335**	**0**.**007**	**−0.566**	**<0.001**
Group 3	**−0.405**	**<0.001**	**−0.980**	**<0.001**	**−0.985**	**<0.001**
Mean difference between groups before confinement						
Between Group 1 and Group 2	**0.238**	**0**.**052**	−0.047	1.000	0.103	0.652
Between Group 1 and Group 3	**0.251**	**0.022**	−0.077	1.000	0.088	0.787
Between Group 2 and Group 3	0.013	1.000	−0.030	1.000	−0.016	1.000
Mean difference between groups during confinement						
Between Group 1 and Group 2	**0.476**	**0**.**001**	0.134	0.929	0.181	0.327
Between Group 1 and Group 3	0.174	0.339	**−0.541**	**<0.001**	**−0.253**	**0**.**051**
Between Group 2 and Group 3	**−0.302**	**0**.**043**	**−0.675**	**<0.001**	**−0.434**	**0**.**001**
Difference in the change of the studied variables between groups						
Between Group 1 and Group 2	**0**.**357**	**0**.**001**	0.044	1.000	0.142	0.245
Between Group 1 and Group 3	0.212	0.060	**−0.309**	**0**.**002**	−0.083	0.837
Between Group 2 and Group 3	−0.144	0.472	**−0.353**	**0**.**002**	**−0.225**	**0**.**027**

Note

Group 1 = Individuals without children; Group 2 = Individuals who spent little or no time with homeschooling; Group 3 = Individuals who spent more time with homeschooling.

### Differences in homeschooling variables between groups: Hypothesis 3

To confirm the hypothesized differences in homeschooling stress and lack of perceived social support from schools by parents due to homeschooling, ANOVA analyses were performed. The results showed significant differences in the level of both homeschooling stress—M_G2_ = 1.47, SD = 0.93; M_G3_ = 2.76, SD = 1.12; *F*(1, 233) = 89.693, *p* < 0.001, η^2^ = 0.278, OP = 1.000—and lack of perceived SSbySchools—M_G2_ = 1.57, SD = 1.02; M_G3_ = 2.46, SD = 1.19; *F*(1, 233) = 36.906, *p* < 0.001, η^2^ = 0.137, OP = 1.000—between G2 and G3. Thus, H3 was supported in that homeschooling stress and lack of perceived social support were higher for G3.

### Direct explanatory role of the variables on stress and anxiety

Correlation analyses showed the expected relationships between variables (correlation table is accessible upon request to the corresponding author). The LRA provided evidence for the direct explanatory role of the studied variables on anxiety and stress (Table 3). Regarding anxiety, for G1, Model 1 explained 67% of the variance, contributing all the variables (age, loneliness, and stress) except gender to the equation. For G2, Model 1 explained 55% of the variance and Model 2 explained 54%; moreover, the introduction of the variables related to homeschooling (lack of perceived social support from schools and homeschooling stress) in Model 2 did not significantly increase the percentage of variance explained by the first model, contributing significantly only loneliness and stress to the equation in Model 2. In contrast, for G3, the introduction of these variables significantly increased the percentage of variance explained by the model (56% for Model 1 and 57% for Model 2), significantly contributing the perceived lack of social support provided by the school, loneliness, and stress to the equation for Model 2.

**TABLE 3:** Impact of study variables on anxiety and stress during confinement

	ß (*p*)			Adjusted *R* ^2^			*F*(*gl*)/*p*			∆*R* ^2^/∆*F* (*p*)		
	G1	G2	G3	G1	G2	G3	G1	G2	G3	G1	G2	G3
1. Stress as dependent variable												
Model 1. Model with variables valid for all participants												
Age	**0**.**13 (0.004)**	0.11 (0.106)	−0.05 (0.417)	0.67	0.55	0.56	**82.773 (4, 160)/<0.001**	**32.475 (4, 99)/<0.001**	**41.8642 (4, 126)/<0.001**	–	–	–
Gender	−0.02 (0.665)	−0.11 (0.105)	−0.06 (0.309)									
Loneliness	**0**.**18 (<0.001)**	**0.20 (0.004)**	**0**.**22 (0.001)**									
Stress	**0**.**75 (<0.001)**	**0**.**66 (<0.001)**	**0**.**64 (0.001)**									
Model 2. Model by adding variables valid only for participants with children												
Age	–	0.12 (0.098)	−0.07 (0.241)	–	0.54	0.57	–	**21.433 (6, 97)/<0.001**	**30.178 (6, 124)/<0.001**	–	0.003/0.286 (0.752)	**0**.**023/3.492 (0.033)**
Gender	–	−0.10 (0.164)	−0.05 (0.430)									
Lack of perceived SSbySchools	–	−0.07 (0.459)	**0**.**19 (0.013)**									
HS stress	–	0.06 (0.532)	−0.03 (0.685)									
Loneliness	–	**0**.**20 (0.005)**	**0**.**23 (<0.001)**									
Stress	–	**0**.**65 (<0.001)**	**0**.**58 (<0.001)**									
2. Anxiety as dependent variable												
Model 1. Model with variables valid for all participants												
Age	**−0.16 (0.001)**	−0.07 (0.329)	0.10 (0.125)	0.65	0.50	0.51	**75.341 (4, 160)/<0.001**	**26.513 (4, 99)/<0.001**	**34.407 (4, 126)/<0.001**	–	–	–
Gender	−0.02 (0.643)	0.11 (0.113)	−0.02 (0.739)									
Loneliness	−0.03 (0.543)	−0.01 (0.904)	−0.01 (0.980)									
Anxiety	**0**.**80 (<0.001)**	**0**.**73 (<0.001)**	**0**.**72 (<0.001)**									
Model 2. Model by adding variables valid only for participants with children												
Age	–	−0.04 (0.557)	0.04 (0.493)	–	0.52	0.60	–	**19.216 (6, 97)/<0.001**	**33.002 (6, 124)/<0.001**	–	0.026/2.748 (0.069)	**0**.**093/14.952 (<0.001)**
Gender	–	0.13 (0.077)	0.01 (0.979)									
Lack of perceived SSbySchools	–	−0.04 (0.679)	0.01 (0.947)									
HS stress	–	0.20 (0.061)	**0**.**35 (<0.001)**									
Loneliness	–	0.01 (0.938)	0.05 (0.468)									
Anxiety	–	**0**.**69 (<0.001)**	**0**.**55 (<0.001)**									

Note

G1 = Individuals without children; G2 = Individuals who spent little or no time with homeschooling; G3 = Individuals who spent more time with homeschooling; HS = homeschooling; SSbySchools = social support provided by teachers and schools’ staff.

Regarding stress, for G1, Model 1 explained 65% of the variance, contributing age and anxiety but not gender and loneliness to the equation. For G2, Model 1 explained 50% of the variance and Model 2 explained 52%, contributing significantly only anxiety to the equation for both models. Thus, for G2, the introduction of the variables related to homeschooling in Model 2 did not significantly increase the percentage of variance explained by the first model, indicating a nonsignificant contribution of those variables. In contrast, for G3, the introduction of those variables significantly increased the percentage of variance explained by the model (51% for Model 1 and 60% for Model 2), contributing significantly both homeschooling stress and anxiety to the equation for Model 2. Thus, H4 (regarding the impact of loneliness on anxiety and the relation between anxiety and stress) and H5 (regarding the impact of the schooling‐at‐home variables on stress and anxiety) were supported.

### Explanatory model: assessment of Hypotheses 4 and 5

**FIGURE 2:**
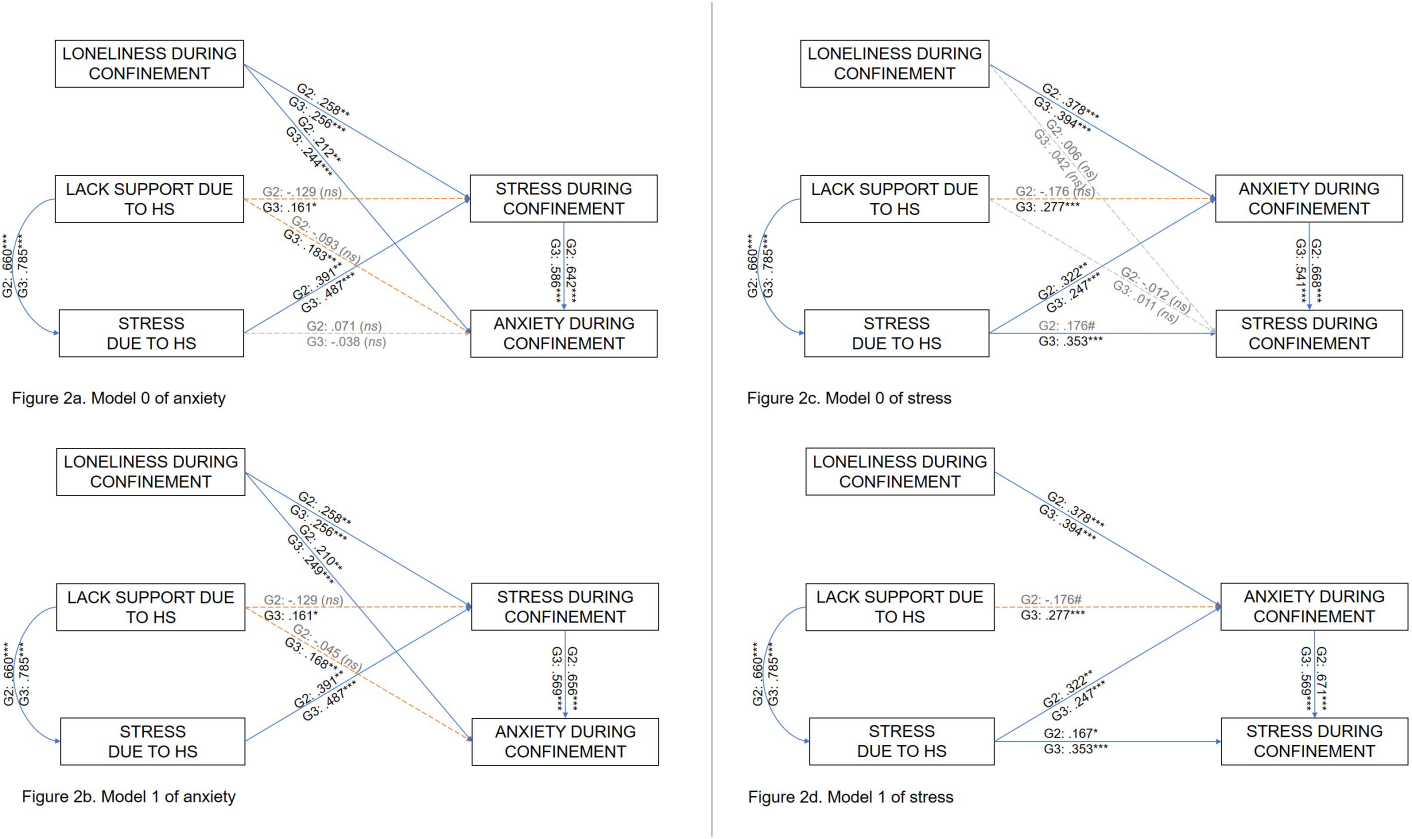
Competing models of anxiety and stress examined in the study. Values represent standardized betas. G2 = individuals who spent little or no time with homeschooling; G3 = individuals who spent more time with homeschooling. The solid blue (dark) lines represent relationships that are significant for both G2 and G3. The dashed orange (dark grey) lines represent relationships that are significant only for G3. The dashed grey (light grey) lines represent relationships that are non‐significant for both G2 and G3

Competing explanatory models for G2 and G3 were examined by performing and comparing different nested and non‐nested multigroup path analyses. Fit indices of all the resulting models (Figure 2) can be observed in Table 4. For both anxiety and stress, the fit indices of Model 0 and Model 1 were excellent. Meanwhile the χ^2^ change showed that for both anxiety and stress the nested models (Model 0 and Model 1) were not significantly different, the fit indices indicated that, for both anxiety and stress, Model 1 fit the data better (lower RMSEA and AIC values) than Model 0. Moreover, when comparing Model 1 of stress and Model 1 of anxiety, the parsimony‐adjusted measures (RMSEA and AIC) showed that the stress model was more parsimonious. Thus, Model 1 of stress (Figure 2d) was the model finally retained.

**TABLE 4:** Fit indices of the competing models

Models	χ^2^ (*df*)	*p*	χ^2^/*df*	GFI	AGFI	CFI	NFI	RMSEA (95% CI)	TLI	AIC	Multigroup ∆χ^2^ (*df*)/*p*	Nested models ∆χ^2^ (*df*)/*p*
Anxiety nested models												
Model 0												
Values for G2	0.583 (2)	0.747	0.291	0.998	0.983	1.000	0.997	0.001 [0.001, 0.135]	1.044	26.583	–	–
Values for G3	1.202 (2)	0.548	0.601	0.996	0.973	1.000	0.995	0.001 [0.001, 0.150]	1.017	27.202	–	
Multigroup unconstrained model (MU)	1.784 (4)	0.775	0.446	0.997	0.977	1.000	0.996	0.001 [0.001, 0.066]	1.028	53.785	MFC→MU: 11.802 (9)/0.107	
Multigroup fully constrained model (MFC)	13.586 (11)	0.257	1.235	0.978	0.939	0.993	0.967	0.032 [0.001, 0.079]	0.988	51.586		
Model 1												
Values for G2	1.106 (3)	0.776	0.369	0.996	0.979	1.000	0.994	0.001 [0.001, 0.110]	1.039	25.106	–	M0→M1: 0.080 (1)/0.777
Values for G3	1.428 (3)	0.699	0.476	0.996	0.978	1.000	0.993	0.001 [0.001, 0.110]	1.022	25.428	–	
Multigroup unconstrained model (MU)	2.533 (6)	0.865	0.422	0.996	0.978	1.000	0.994	0.001 [0.001, 0.045]	1.029	50.533	MFC→MU: 11.133 (6)/0.084	
Multigroup fully constrained model (MFC)	13.666 (12)	0.323	1.139	0.977	0.944	0.996	0.967	0.024 [0.001, 0.073]	0.993	49.666		
Stress nested models												
Model 0												
Values for G2	0.583 (2)	0.747	0.291	0.998	0.983	1.000	0.997	0.001 [0.001, 0.135]	1.044	26.583	–	–
Values for G3	1.202 (2)	0.548	0.601	0.996	0.973	1.000	0.995	0.001 [0.001, 0.150]	1.017	27.202	–	
Multigroup unconstrained model (MU)	1.784 (4)	0.775	0.446	0.997	0.977	1.000	0.996	0.001 [0.001, 0.066]	1.028	53.784	MFC→MU: 14.972 (7)/0.036	
Multigroup fully constrained model (MFC)	16.756 (11)	0.115	1.523	0.973	0.925	0.986	0.960	0.047 [0.001, 0.090]	0.974	54.756		
Model 1												
Values for G2	0.604 (4)	0.963	0.151	0.998	0.991	1.000	0.996	0.001 [0.001, 0.001]	1.053	22.604	–	M0→M1: 0.365 (2)/0.833
Values for G3	1.684 (4)	0.794	0.421	0.995	0.981	1.000	0.993	0.001 [0.001, 0.085]	1.025	23.683	–	
Multigroup unconstrained model (MU)	2.286 (8)	0.971	0.286	0.996	0.985	1.000	0.995	0.001 [0.001, 0.001]	1.036	46.286	MFC→MU: 14.835 (8)/0.011	
Multigroup fully constrained model (MFC)	17.121 (13)	0.194	1.317	0.972	0.934	0.990	0.959	0.037 [0.001, 0.079]	0.984	51.121		

Note

G2 = Individuals who spent little or no time with homeschooling; G3 = Individuals who spent more time with homeschooling.

As seen in Table 4, the results of the χ^2^ change between the unconstrained and fully constrained models of stress showed that G2 and G3 were different at the model level. When comparing G2 and G3 at the path level in Model 1 of stress by using the critical ratio for differences between parameters, significant differences were observed in the link between lack of SSbySchools and anxiety during confinement (*z* score = −2.899, *p* < 0.001); this path was significant for G3 but not for G2 (see Figure 2d). And even if the paths between anxiety and stress were both significant, a significant difference was found, showing that the relation between those two variables was stronger for G3 than for G2. Thus, H4 (regarding the impact of the schooling‐at‐home variables on stress and anxiety) and H5 (the impact of the homeschooling variables on stress and anxiety) were supported. Nevertheless, for G2 the lack of social support by schools did not explain the anxiety.

## DISCUSSION

The sudden onset of the COVID‐19 pandemic around the globe has challenged our understanding of parental well‐being, given that never before have so many parents and their children been confined to continue work and school from home. This study examined the impact of homeschooling in a Spanish context due to a stay‐at‐home order on the mental health outcomes of parents with school‐age children. The main objectives were to compare the differential impact of COVID‐19 confinement on mental outcomes (loneliness, anxiety, and stress) in individuals without children at home and in parents who spent little versus more time on homeschooling and to explore the role played by loneliness and appraisal of discomfort due to homeschooling (perceived SSbySchools and homeschooling stress) in the rates of stress and anxiety of parents during confinement.

### Loneliness, anxiety, and stress in confined individuals

The results provide evidence for the impact of confinement on mental health, with the rates of all the studied variables found to be significantly more elevated during than before confinement, as other studies have shown (Brooks et al., 2020; Tull et al., 2020).

When looking at the three observed groups regarding perception of anxiety and stress, individuals in all three groups experienced significant increases during confinement. Furthermore, differences were found between the groups. As expected, for parents who spent more time dedicated to homeschooling, the perception of anxiety and stress rates were higher than for parents who spent little or no time dedicated to homeschooling and individuals without children at home.

However, no differences were found between people without children at home and those with children at home who spent little time dedicated to homeschooling. Thus, framed in the definition of stress (Folkman, 2013), the results were congruent with the argument that forced homeschooling dedication could act as an additional stressor for parents; parents have had to adopt a new role for which they were not prepared, by assuming primary responsibility for their children's learning (Doyle, 2020). Thus, they may perceive that they do not have sufficient resources to face the new and unexpected homeschooling situation, under stress, during confinement. Consequently, their stress and anxiety levels may be acute in comparison to individuals without children at home and parents who dedicate little time to homeschooling. These results are especially relevant for mental health and argue for the need to pay special attention to those parents who dedicate extra time to homeschooling in psychological interventions. In this sense, both teachers and health professionals should assist parents who are involved with homeschooling, maybe by developing parent support forums, virtual groups, or lists of resources for improving their mental health.

We were also interested in studying the evolution of the perception of loneliness between before and during the confinement. In line with previous studies (Röhr et al., 2020; Tull et al., 2020), the increases were significant for individuals without children at home and for parents who dedicated more time to homeschooling. Nevertheless, unexpectedly, for parents who spent little or no time on homeschooling, their perception of loneliness levels did not show any change.

Moreover, the expected differences between groups in the loneliness increase were only partially supported: as expected, loneliness levels perceived before confinement were higher in individuals without children at home than in the other two groups, in agreement with previous studies affirming that loneliness is lower in individuals with children (Beutel et al., 2017). Nevertheless, even if, as expected, levels of loneliness during the confinement were higher for people without children at home than in parents who spent little time dedicated to homeschooling, unexpectedly, no differences were found between people without children at home and parents who dedicated more time to homeschooling. In consequence, the levels of loneliness experienced during the confinement of parents who dedicated little time to homeschooling were significantly lower than in individuals without children at home (as expected), but also lower than in individuals who dedicated more time to homeschooling.

These results may be explained by the fact that having children at home—but not being overwhelmed with homeschooling tasks that may enhance the feeling of helplessness—could protect individuals from the implications of confinement on loneliness. Framed in the theory of belongingness (Baumeister & Leary, 1995), we argue that for individuals who have been forced to isolate themselves, having children at home may fulfill the need for belongingness, and thus loneliness could be prevented. However, for parents who spend more time dedicated to homeschooling this effect seems to disappear, maybe because homeschooling makes them feel that they need more social support to cope effectively with homeschooling, and then this feeling of lack of social support makes them feel lonely. They could also feel unprotected and helpless, needing more support to cope effectively with homeschooling, and such perceived helplessness may make them feel lonelier in the face of this adversity. In this sense, previous studies have demonstrated that helplessness is related to loneliness (Vasileiou et al., 2017). More studies are needed to explore whether the differences in the loneliness levels perceived by parents devoting more versus less time to homeschooling could be due to a perception of helplessness.

### Homeschooling as a stressful situation

Homeschooling was perceived as a stressful situation. The new homeschooling situation implies modification in the teaching and family–teacher communication processes (Doyle, 2020; Verma et al., 2020) and in the role of parents (who are not necessarily prepared) as the prime drivers of learning (Doyle, 2020). Thus, both the perception of SSbySchools and homeschooling stress may be affected. As expected, parents who spent more versus less time on homeschooling experienced more homeschooling stress and perceived less SSbySchools. These results are relevant because the appraisal of discomfort due to homeschooling may be construed as a lack of resources to cope effectively with stress in the confinement situation, resulting in higher stress and anxiety.

### Relations among variables in explaining stress and anxiety during confinement

The different analyses performed provided evidence for the expected relations among variables. In accordance with previous studies that have related loneliness (Beutel et al., 2017; Kara et al., 2019; Muyan et al., 2016; Zawadzki et al., 2013) and stress (Campos et al., 2013; McEwen et al., 2012; Zvolensky et al., 2002) to anxiety, the loneliness and stress experienced during confinement were associated with anxiety for all three groups. Moreover, integration of the variables related to homeschooling significantly increased the percentage of variance explained by the other variables in parents who dedicated more time to homeschooling but not in those who dedicated little time to it. In fact, for parents with more dedication to homeschooling, but not for those with less, the perceived lack of SSbySchools significantly explained anxiety. A similar pattern occurred with stress: in accordance with previous studies that had related anxiety (Fawzy & Hamed, 2017; Gillott & Standen, 2007; Phillips et al., 2015) to stress, in our study the anxiety experienced during confinement significantly explained the stress, and integration of the variables related to homeschooling significantly increased the percentage of variance explained by the other variables in parents who dedicated more time to homeschooling, versus less. This time, homeschooling stress was the variable that significantly explained the stress for parents with more homeschooling dedication but not for parents with less, for whom the relation was only marginal.

These results were supported in the explanatory models explored, in which the lack of SSbySchools was relevant in the explanation of anxiety and stress during confinement only when parents dedicated more time to homeschooling. In contrast, when dedication to homeschooling was limited, the perceived SSbySchools was not relevant for anxiety during confinement (nor for stress in the anxiety model). Regarding homeschooling stress, this was relevant in the explanation of stress for parents who dedicated both little and more time to homeschooling. Then, another practical implication is framed in the schooling context. At this time, the effect of COVID‐19 on future education is uncertain: in the near term, it is not known whether students will return to school or learn remotely or partially online. If homeschooling is fully or partially maintained, educators and schools may wish to emphasize the social support provided to families because this could affect the quality of student learning and the mental health of parents, and thus indirectly children's well‐being.

In a forced homeschooling situation, responsibility for the learning processes must not fall excessively or exclusively on parents. Teachers must still be the principal drivers of the learning processes, and they must accompany and support parents, thus requiring resources from the government and schools. Moreover, our results supported the idea that the less parents perceive SSbySchools, the more they dedicate time to homeschooling. For parents who dedicated less time to homeschooling, their perception of SSbySchools was probably not too low, and thus it did not explain their mental health. In contrast, for parents who dedicated more time, the perception of social support was directly related to mental health. Thus, this variable could be relevant in protecting parents from anxiety and subsequently from stress.

Because of the cross‐sectional nature of our study, conclusions about causation and direction cannot be made, but the results suggest that both directions are plausible; the literature agrees with this affirmation, with studies demonstrating both stress‐induced anxiety (Campos et al., 2013; McEwen et al., 2012; Zvolensky et al., 2002) and anxiety‐induced stress (Fawzy & Hamed, 2017; Gillott & Standen, 2007; Phillips et al., 2015). Our results suggest that two‐way feedback is possible. Furthermore, the results imply that psychologists must attend to the loneliness levels experienced during confinement because they affect mental health outcomes and are strongly related to parental stress and anxiety experienced during confinement. Moreover, for parents with children at home who dedicate much time to homeschooling, health professionals should also attend to the discomfort with homeschooling, which seems to be related to their stress and anxiety.

### Limitations and future research

Although the results of this study are promising, some limitations must be highlighted. The major limitation is probably the sampling procedure. Sharing the questionnaire with social networks (chosen due to the State of Alarm in Spain) produced a disproportionately high number of participants from Andalusia, a region with more than 8 million citizens and where residents show high identity as Spaniards and Andalusians (Huici et al., 2008). Moreover, differences were found in the percentages of Andalusian and Castilian‐Leonese in the three compared groups. Thus, the generalization of results should be done with caution, and the different proportions of residents from different regions and their unequal incidence of COVID‐19 in each group may have affected the results. Similarly, the results of this study refer to families that have not chosen homeschooling because this option is not legal in Spain; thus the generalization of results to other countries should be done with caution, especially in those countries where homeschooling is regulated and thus could be freely elected by some families. Nevertheless, there is no reason to think that people of other countries who have not chosen homeschooling should process and manage their mental health differently when faced with forced homeschooling and confinement.

Another limitation refers to the sample discrimination. More data could have been gathered from respondents in all three groups about the amount of time working, the kind of job, and the socioeconomic situation. Also, more information such as age and school grades, the potential help provided at home (e.g., older family members), technology and Internet access, etc., could be relevant. Those sociodemographic variables might have affected the relationships among the key study variables and should be analyzed in the future.

However, it can be observed that, as a whole, the sociodemographic variables were similar in the three groups. Regarding the job situation, a similar percentage of participants was working in each of the three groups. Nevertheless, the percentage of jobless participants was higher for participants without children at home than for participants with children at home who dedicated more time to homeschooling. In this sense, as obviously expected, in the group without children at home there were more retired people than in the other two groups. Nevertheless, the number of retired people was not high enough to include this variable in the analyses. Differences between groups were also observed in terms of age: individuals with children at home who dedicated more time to homeschooling were significantly older than individuals in the other two groups. Moreover, participants without children at home reported having no partners more frequently than participants with children at home, regardless of their dedication to homeschooling. Also, as expected, individuals with no children at home reported sharing their homes with fewer individuals than the other groups. Finally, individuals who dedicated more time to homeschooling reported having more children than individuals who dedicated little or no time to homeschooling. Again, in future research and with a greater and more heterogeneous sample, it would be interesting to analyze how the sociodemographic variables could influence the relationships observed in this study, as they might have partially explained the study findings.

Another limitation refers to the fact that levels of the studied variables before the confinement were collected from retrospective report. Then, results about the evolution of the variables should be interpreted with caution. Finally, depression was not assessed. However, given the associations of all of our variables related to distress and lockdowns consequent to COVID‐19 with depressive symptoms and diagnoses, future research is needed on the relation between lockdown and depression by comparing parents with and without children at home and also their dedication to homeschooling. Similar differences to those found between groups may be found in the depression levels and also similar associations between variables. Future studies could benefit by including depression as a dependent variable and comparing the different associations between variables for the observed groups.

## CONCLUSIONS

This study explored the differential impact from confinement due to COVID‐19 on the mental health of individuals without children at home and parents who dedicated little versus more time to homeschooling. Anxiety and stress perceptions increased in the three observed groups during confinement, providing evidence for the impact of the confinement on health. Moreover, this effect was higher for parents who dedicated more time to homeschooling than for parents who dedicated less time and also for individuals without children at home. Additionally, with regard to loneliness, parents who dedicated little time to homeschooling were protected from the confinement effect, whereas the other two groups suffered an increase in loneliness. These results suggest that homeschooling is associated with the negative effect that confinement has on mental health outcomes. Moreover, parents who dedicated more time to homeschooling felt unprotected due to the confinement and stressed due to homeschooling compared to parents who dedicated little time to homeschooling. Finally, loneliness and discomfort due to homeschooling explained the anxiety and stress levels suffered by parents during confinement, with the variables related to homeschooling particularly relevant for parents who dedicated more time to homeschooling.

The significance of this study relies both on the fact that the relation of homeschooling due to COVID‐19 with parental mental health outcomes has not been explored before, as well as findings about the differential impact of confinement in the three observed groups. The major implications are that health professionals must pay special attention to parents who dedicate more time to homeschooling due to pandemic confinement. Moreover, the government, health services, and schools must emphasize social support provision for families in a forced homeschooling situation.

## CONFLICT OF INTEREST

The authors report no conflict of interest.
